# Foresight in a Game of Leadership

**DOI:** 10.1038/s41598-020-57562-1

**Published:** 2020-02-10

**Authors:** Logan Perry, Sergey Gavrilets

**Affiliations:** 1 Department of Mathematics, Center for the Dynamics of Social Complexity, University of Tennessee, Knoxville, TN 37996 USA; 2 Department of Ecology and Evolutionary Biology, National Institute for Mathematical and Biological Synthesis, University of Tennessee, Knoxville, TN 37996 USA

**Keywords:** Cultural evolution, Evolutionary theory, Social evolution

## Abstract

Leadership can be effective in promoting cooperation within a group, but as the saying goes “heavy is the head that wears the crown”. A lot of debate still surrounds exactly what motivates individuals to expend the effort necessary to lead their groupmates. Evolutionary game theoretic models represent individual’s thought processes by strategy update protocols. The most common of these are random mutation, individual learning, selective imitation, and myopic optimization. Recently we introduced a new strategy update protocol - foresight - which takes into account future payoffs, and how groupmates respond to one’s own strategies. Here we apply our approach to a new 2 × 2 game, where one player, a leader, ensures via inspection and punishment that the other player, a subordinate, produces collective good. We compare the levels of inspection and production predicted by Nash Equilibrium, Quantal Response Equilibrium, level-k cognition, fictitious play, reinforcement learning, selective payoff-biased imitation, and foresight. We show that only foresight and selective imitation are effective at promoting contribution by the subordinate and inspection and punishment by the leader. The role of selective imitation in cultural and social evolution is well appreciated. In line with our prior findings, foresight is a viable alternative route to cooperation.

## Introduction

The web of social and economic ties that weaves us together has never been thicker. Every day we make decisions based on our expectations of how others will act^1^. While these waters are difficult to traverse, we are not without aid as there exists a plethora of mores to help us navigate this interpersonal maelstrom. The mores that coordinate political and economic relationships are referred to as institutions^2–4^. Whether it is a teacher corralling their students’ behavior or the United Nations placing sanctions on an entire nation, institutions serve as an effective tool for resolving conflict and shaping behavior. The role of institutions in modern society cannot be understated, with some claiming they are the determining factor of whether nations succeed or fail^3,5^. However, their origins are much older than modern society as they existed even in hunter-gather groups^6^.

Here, we are concerned with the institution of leadership in small-scale societies^7,8^. A leader is defined as an individual who has non-random differential influence over group behavior^9^. Leaders can take on several different roles within a group, e.g. role-models^10^, managers^11^, punishers^12^ or volunteers^13^. Leader-follower relationships are likely to emerge in groups of conspecifics that benefit from acting in a unified manner^14^. Examples of actions that necessitate high degrees of coordination include migration, hunting, deterring predation, resolving internal conflicts, and competing with neighboring groups^14^.

Joint actions often lead to the collective action problem^15^ when all individuals can benefit from an action but no one is willing to bear its cost. The collective action problem (CAP) is present in many animal and human groups^15–19^. Several solutions to the CAP exist. They include kin selection, direct and indirect reciprocity, punishment^20^, selective incentives and institutional design^15,21^, the presence of within-group heterogeneity^17,22–24^ and influential individuals^25^.

Here we are focused on the solution by way of punishment and the institution of leadership. In particular, we are concerned with the question of why leaders would choose to enforce cooperation since abstaining from doing it could be less costly - the so called second-order free-rider effect. We investigate this by developing a modified inspection game^26^.

Inspection games are typically concerned with modeling an *inspector* that seeks to verify the adherence to some pre-arranged contract and an *inspectee* that may be tempted to violate said contract. In the past, inspection games have been used to better understand relationships between law enforcement and criminals^27–30^, employers and their employees^31^, and countries dealing with nuclear armament^32^. Here we modify the standard inspection game^26^ to account for interactions between a leader and a subordinate. We do this by altering basic assumptions so that the subordinate has no incentive to produce a good, while the leader has a vested interest in seeing the good produced. Our motivation for doing this is to mirror a standard CAP with punishment in a two person game. In particular we are interested in our model having properties that parallel the first- and second-order free-rider effects. The simplicity of our proposed model allows us the opportunity to obtain analytical results, which can in turn be used to provide a baseline understanding of more complicated models.

To accurately portray the dynamics of the leader-subordinate relationship we must consider their thought processes. While both individuals are interested in bettering their own positions, the leader has sway over the subordinate and must make decisions based on this influence. This means that the leader must anticipate how their subordinate will react and hence must take into consideration how their subordinate makes decisions. This consideration of other’s cognitive processes is referred to as a “theory of mind”^33,34^. A “theory of mind” is thought to be key to promoting cooperation withing groups^34^, has been linked to the size of individuals’ social networks^35^, their propensity for social cooperation^36^, and the extent to which they are agreeable^37^. Standard approaches of myopic optimization, adjustment through error and mutation, reinforced learning, and selective imitation fail to account for this theory. Here we continue to develop the strategy update protocol of foresight^38^, which, we argue, incorporates some aspects of the theory of mind and in doing so solves the second-order free-rider effect.

Foresight works by altering agents’ utility functions to consider not only how a strategy will do in this round, but how it will affect the next round as well^38^. Fundamental to foresight is the fact that agents consider how their actions will shape the future behaviors of others. Thus it accounts both for human’s “theory of mind” and the “shadow of the future”^39^. The major focus of our earlier work was the impact of foresight on a population facing a collective action problem in the presence of peer punishment. We showed that in heterogeneous groups possessing foresight a division of labor will develop where the strong will specialize in enforcing the contributions of the weak. We now seek to further develop our theory of foresight and obtain analytical results.

Our approach here is broken into several phases. We begin by developing a two-player game based on the classical inspection game^26^ to investigate the relationship between a leader and their subordinate, which we dub the leadership game. As in our original paper^38^, we assume heterogeneity, but now take it to be systemic. This is done by pre-designating a leader who serves as an enforcer over a subordinate. We show that the leader lacks motivation to enforce the contribution of their subordinate despite their vested interests in seeing goods produced. Our investigation of the leadership game is structured as follows. First, we consider some typical approaches to analyzing the leadership game, e.g. Nash equilibria, Quantal Response Equilibria (QRE) models, level-*k* models, and fictitious play models. We follow this up by investigating the notion of foresight, which we previously introduced^38^. Our results show that the ability of foresight successfully motivates leaders to punish subordinates, and in turn motivates subordinates to produce. Finally, we compare and contrast foresight with two different learning protocols: Cross’s reinforcment learning model^40^ and selective imitation^41^.

## Results

### Leadership game

We consider a simple 2 × 2 game played between a leader and a subordinate, which is based on the inspection game^26^ described in the Supplementary Information (SI). The subordinate is tasked with producing a good or benefit at a personal cost to themselves, while the leader has a vested interest in seeing the good is produced. Since we are interested in drawing parallels with collective action problems we make assumptions in such a way that the subordinate has no incentive to see the good be produced unless they are facing punishment.

The subordinate can either produce the good ($x=1$) or shirk on the production of the good ($x=0$). In the case the subordinate produces the good, they pay a cost of *c* to produce a good of value *b*. Any benefit produced by the subordinate is split with the leader in a $\theta :1-\theta$ ratio. Here $0\le \theta \le 1$ can be thought of as a taxation rate. The strategies available to the leader are to enforce production via inspection ($y=1$) or to not inspect ($y=0$). Inspection costs the leader *h*, but in the event that a leader inspects a non-producing subordinate they inflict a punishment of *d* at a cost of *k*. We assume that all parameters are positive (see Table 1). Table 2 describes the corresponding payoff matrix.

**Table 1:** Parameters in the leadership game.

Parameter	Interpretation
*b*	Benefit produced by a subordinate
*c*	Cost of contributing to a subordinate
*d*	Punishment for shirking
*h*	Cost of inspecting to a leader
*k*	Cost of punishing to a leader
*θ*	Taxation rate
*λ*	Precision parameter
*ω*	Foresight parameter

**Table 2:** Payoff matrix for the basic leadership game.

	Leader		
Subordinate		Inspect	Don’t Inspect
	Produce	$(1-\theta )b-c$, $\theta b-h$	$(1-\theta )b-c$, *θb*
	Shirk	−*d*, $-h-k$	0, 0

The payoff functions for the leader and the subordinate are then

1a $${\pi }_{S}(x,y)=[(1-\theta )b-c]x-dy(1-x),$$

1b $${\pi }_{L}(x,y)=b\theta x-[h+k(1-x)]y.$$

We will make three assumptions. First, given the subordinate contributes (i.e., $x=1$), the benefit to the leader exceeds its cost of inspection, i.e., $\theta b > h$. Second, given our aforementioned interest in mirroring CAP in a two-person game, we assume that without punishment (i.e., if $y=0$) the subordinate is not motivated to contribute, i.e., $(1-\theta )b-c < 0$. Third, facing the threat of punishment (i.e., if $y=1$), the subordinate however is motivated to contribute, i.e., $(1-\theta )b-c > -\,d$.

#### Pure strategies

Next we derive best response functions for the subordinate, ${{\rm{BR}}}_{S}(y)$, given the leader’s action *y* and for the leader, ${{\rm{BR}}}_{L}(x)$, given the subordinate’s action *x*. If the leader inspects ($y=1$), the subordinate prefers to produce ($x=1$). If, however, the leader doesn’t inspects, then the subordinate’s best option is to do nothing. Therefore, ${{\rm{BR}}}_{S}(y)=y$. This implies that the subordinate can be motivated to produce the good. On the other hand, the leader’s best response is not to inspect no matter what the subordinate does: ${{\rm{BR}}}_{L}(x)=0$. Therefore, the only Nash equilibrium is $({x}^{\ast },{y}^{\ast })=(0,0)$.

#### Mixed strategies

Suppose that the subordinate chooses to produce the good with probability *p* while the leader opts to inspect with probability *q*. Then the expected payoffs are

2a $${E}_{S}(p,q)=p[(1-\theta )b-c]-(1-p)qd,$$

2b $${E}_{L}(p,q)=p\theta b-q[h+(1-p)k].$$

From the above we can see that the subordinates best response depends upon the leaders strategy. In particular, there is a critical inspection rate of the leader,

3 $${q}_{c}=\frac{c-(1-\theta )b}{d},$$

(0 ≤ *q*_*c*_ ≤ 1), such that if $q < {q}_{c}$, the subordinate is best off always doing nothing: $B{R}_{S}(q)=0$. If $q > {q}_{c}$, the subordinate is best off always producing the good: $B{R}_{S}(q)=1$. If $q={q}_{c}$, the subordinate will receive the same payoff no matter what they do. The case of the leader is much simpler, as the leader’s best response is always to do nothing: $B{R}_{L}(p)\equiv 0$. Hence, the only Nash equilibrium is at $({p}^{\ast },{q}^{\ast })=(0,0)$ when the subordinate does not contribute and the leader does not inspect.

#### Implications for evolutionary dynamics

Assume that individuals are bounded rational and attempt to increase their payoffs by evaluating some “candidate” strategies, which they generate mentally, and choosing one with probabilities proportional to estimated payoffs. (In the terminology of ref. ^42^, this is a direct strategy revision protocol). The results above imply that the game will converge to the Nash equilibrium (0, 0) of nothing being done.

### Quantal Response Equilibrium (QRE)

Next, we generalize our results for the case when agents make errors in evaluating payoffs. We do this by relaxing the assumption that individuals are best responders and replace it with the assumption that individuals are better responders. In this new paradigm, all strategies are played with non-zero probabilities, but the rate at which they are played is proportional to their payoff. This approach leads to investigate what is known as the Quantal Response Equilibrium^43^ of our model.

Let *p* be the probability the subordinate contributes, and *q* be the probability the leader inspects. Let ${E}_{S,0}={\pi }_{S}(1,q)$ and ${E}_{S,1}={\pi }_{S}(0,q)$ be the expected payoff of not contributing and contributing to the subordinate, respectively. Define ${E}_{L,0}={\pi }_{L}(p,0)$ and ${E}_{L,1}={\pi }_{L}(p,1)$ for the leader similarly. In the QRE approach, *p* and *q* are specified as

4 $$p=\frac{1}{1+\exp [\lambda ({E}_{S,0}-{E}_{S,1})]},\,q=\frac{1}{1+\exp [\lambda ({E}_{L,0}-{E}_{L,1})]},$$

where *λ* is the precision parameter (e.g., with $\lambda =0$, the players’ decision are random: $p=q=0.5$, while as $\lambda \to \infty$, both player use myopic best response). Note that we assume both players have the same precision. The QRE solutions for *p* and *q* satisfy the equalities^43^

5 $$\frac{\mathrm{ln}(p(1-p))}{\lambda }={E}_{S,0}-{E}_{S,1},\,\frac{\mathrm{ln}(q(1-q))}{\lambda }={E}_{L,0}-{E}_{L,1}.$$

We solve the above equations numerically. Figure S1 in the SI shows the impact that the precision parameter *λ* has on the QRE values $({p}^{\ast },{q}^{\ast })$. For $\lambda =0$ play is perfectly random so that ${p}^{\ast }={q}^{\ast }=\frac{1}{2}$, as we would expect. As we increase *λ*, play converges to the single Nash equilibrium at (0, 0), again as we would expect. So unless error is very high (i.e., *λ* is small), there will be not much inspection or contribution.

Before introducing foresight, we show next that just incorporating a theory of mind in our model is not enough to overcome the free-rider-like effects. We consider two different models attempting to capture some aspects of this theory.

### Level-*k* approach

A common way for capturing a theory of mind in models of decision-making is by supposing the agents utilize level-*k* rationality, which is a hierarchical way of thinking based on iterative logic^44^. In it the most basic model of cognition is level-0 rationality, which makes agents to just play the strategies available to them at random. It is important to note, that players are never assumed to be level-0, but rather it is the simplest model of others a player may have. A level-1 player will assume that all other individuals are level-0 and select a strategy which best responds to their predicted actions. Likewise, a level-2 player will assume all others are level-1 and select a strategy accordingly. In this way we can iteratively define a level-*k* player who will assume all others are level-*k* − 1 and choose a strategy which best responds to their predicted action.

We begin by supposing both agents are level-1, which means they assume the other is level-0. Generalizing slightly, we suppose that a level-0 subordinate is assumed to contribute with probability *p*_0_, while a level-0 leader is assumed to contribute with probability *q*_0_ (e.g. ${p}_{0}={q}_{0}=0.50$). Now the expected payoffs are given by ${E}_{S}(p,{q}_{0})$ and ${E}_{L}({p}_{0},q)$ (See Eqs. (2a) and (2b)). This implies the best response for a subordinate is to not contribute provided the expected cost of being punished is less than the net cost of producing, i.e. ${q}_{0}d < c-(1-\theta )b$. Likewise, it is best to contribute provided the expected cost of being punished is greater than the net cost of producing. Meanwhile the leader’s expected payoff ${E}_{L}({p}_{0},q)$ is always maximized by setting $q=0$ implying it is always best for the leader to not inspect.

Now a level-2 subordinate will assume that the leader is level-1, and thus anticipates that the leader will never inspect. This in turn means that a level-2 subordinate will never contribute. A level-2 leader on the other hand will expect a subordinate to contribute sometimes, but will always be better off doing nothing. So a level-2 leader will always opt to not inspect. Finally, for levels 3 onward we have by similar logic that neither player will do anything. Hence, level-k modeling is unable to overcome the free-rider-like problem in our model.

### Fictitious play

In the model of fictitious play^45^, every player assumes their opponents are playing strategies drawn from a certain stationary distribution which the player attempts to estimate via observation. Each player then chooses their action (i.e., a value of *x* or *y*) in an attempt to maximize their payoff given a prediction or assessment of their opponent’s strategy.

The leader assumes that the subordinate uses a mixed strategy contributing with a certain probability. Let $\tilde{p}(t)$ be the leader’s estimation of this probability at time *t*. The subordinate assumes that the leader uses a mixed strategy inspecting with a certain probability. Let $\tilde{q}(t)$ be the subordinate’s estimation of this probability at time *t*. We take $\tilde{p}\mathrm{(0)}$, and $\tilde{q}\mathrm{(0)}$ to be the initial beliefs. Let $x(t)$ and $y(t)$ be the action taken, i.e. 0 or 1, by the subordinate and leader, respectively, in round *t*. Now we define a system of recurrence equations describing how the leader and subordinate adjust their believes based on observations of previous actions

6a $$\tilde{p}(t+1)=(1-\ell )\tilde{p}(t)+\ell x(t),$$

6b $$\tilde{q}(t+1)=(1-\ell )\tilde{q}(t)+\ell y(t).$$

Here, $\ell$ is a parameter which scales the impact of the most recent action on the agent’s estimation. In general, $\ell$ can depend on *t*. For example, $\ell =\frac{1}{1+t}$ corresponds to the original approach^45^. The case of $\ell =1$ corresponds to best response.

Fictitious play itself is then defined as any rule the agent uses to choose a response from the set of best responses to his or her estimation of the opponent’s strategy. For our case, the natural choice of the rule is given by the best response functions $B{R}_{S}(q)$ and $B{R}_{L}(p)$ established above. Since our game is dominance solvable via iteration (see the SI for details), we know from ref. ^46^ that it will converge to an equilibrium asymptotically. As it has only one Nash equilibrium at (0, 0), it will converge to it. This makes sense as a rational leader would never choose to inspect as not inspecting offers a higher payoff in all circumstances, and a rational subordinate would quickly learn this is the case and thus choose to not contribute. Hence, fictitious play modeling is unable to overcome the free-rider-like problem in our model.

### Foresight

We have shown that under myopic optimization, level-*k* modeling, or fictitious play leaders will fail to enforce and subordinates will fail to contribute. One method to overcoming this is to introduce foresight^38^. If we assume that the leader is willing to suffer a cost this round in order to make a gain in future rounds, then they could be motivated to inspect the subordinate. More specifically, we introduce the foresight parameter $\omega \in (0,1)$, which measures the weight placed on next round’s *forecasted* payoffs versus this round’s anticipated payoff. This averaging of payoff now with payoff later can be compared with the typical practice of discounting future payoffs. Where foresight is particularly novel is how we account for the leader forecasting future payoffs. These forecasted payoffs depend upon the leader’s *model* of their subordinate. This consideration of how their subordinate reasons is where our leader’s theory of mind is on display. We assume that the leader’s model of their subordinate’s behavior is based on a best response and focus on the effect foresight has on a leader’s strategy selection.

We will assume that only the leaders use foresight. (The SI shows that allowing for the subordinate to use foresight does not change our conclusions). Consider a weighted sum of the leader’s payoffs for this and the next rounds:

$$(1-\omega )\times (\theta bx-[h+k(1-x)]y)+\omega \times (\theta b\tilde{x}-[h+k(1-\tilde{x})]\tilde{y}),$$

where $\tilde{x}$ and $\tilde{y}$ are the subordinate and leader’s efforts in the next round. The leader expects that their action this round *y* will affect the subordinate’s action $\tilde{x}$ in the next round. If the subordinate uses best response, as we will assume, $\tilde{x}=y$. At the same time, *y* has no effect on the benefit to be produced by the subordinate this round, *θbx*, or the cost of the inspection in the next round, $[h+k(1-\tilde{x})]y^{\prime}$. Therefore the can define the leader’s utility function as a weighted sum of the costs of inspection and punishment this round and the benefit next round

7 $${u}_{L}(x,y)=(1-\omega )\times (\,-\,[h+k(1-x)]y)+\omega \times (\theta by).$$

The leader’s utility function is thus different from their actual payoffs. For the subordinate who uses best response, the utility function is equal to the expected payoff given by Eq. (1a). Table 3 defines the utilities of different actions in this model.

**Table 3:** Utility matrix for the leadership game with foresight.

	Leader		
Subordinate		Inspect	Don’t Inspect
	Produce	$(1-\theta )b-c$, $-(1-\omega )h+\omega \theta b$	$(1-\theta )b-c$, 0
	Shirk	−*d*, $-(1-\omega )\,(h+k)+\omega \theta b$	0, 0

#### Pure strategies

We can see that the state (0, 0) is still a Nash equilibrium but only if $(1-\omega )\,(h+k) > \omega \theta b$, that is, if the foresight parameter is small enough: $\omega  < {\omega }^{\ast }\equiv \frac{h+k}{\theta b+h+k}$. However there is a new possibility: if $\omega \theta b > (1-\omega )h$, or, equivalently, if the foresight parameter is large enough: $\omega  > {\omega }^{\ast \ast }\equiv \frac{h}{\theta b+h}$, there is another Nash equilibrium (1, 1). For intermediate values of $\omega$, i.e. if ${\omega }^{\ast \ast } < \omega  < {\omega }^{\ast }$, these two equilibria coexist. The leader does the best at the (1, 1) equilibrium where the payoffs are ${u}_{S}=(1-\theta )b-c$ and ${u}_{L}=\theta b-h$. The subordinate does the best at the (0, 0) equilibrium where the payoffs are 0 and 0.

#### Mixed strategies

Assume that the two players make efforts with probabilities *p* and *q*, respectively, and consider formally the corresponding expected utilities:

8a $${U}_{S}(p,q)=[(1-\theta )b-c]p-dq(1-p),$$

8b $${U}_{L}(p,q)=(1-\omega )\,(\,-\,(h+k(1-p))q)+\omega \theta bq.$$

Computing the derivative $\frac{\partial {U}_{S}(p,q)}{\partial p}$, one finds that the subordinate’s utility ${u}_{S}$ increases with $p$ for $q > {q}_{c}$ and decrease otherwise, where *q*_*c*_ is defined by Eq. (3). Similarly, computing the derivative $\frac{\partial {U}_{L}(p,q)}{\partial q}$, the leader’s utility increases with *q* if $p > {p}_{c}$ and decreases otherwise. The critical value

9 $${p}_{c}=\frac{1}{k}(h+k-\frac{\omega \theta b}{1-\omega }).$$

Note that ${p}_{c} < 0$ and thus the condition $p > {p}_{c}$ is always satisfied if $\omega \theta b > (1-\omega )(h+k)$, i.e., if the expected future benefit is larger than the current cost of inspection and punishment (or, equivalently, if $\omega  > {\omega }^{\ast \ast }$). There is a mixed Nash equilibrium $({p}^{\ast }={p}_{c},{q}^{\ast }={q}_{c})$, but this equilibrium is unstable: if one player deviates from it, the other player will be motivated to change their strategy as well.

#### Implications for evolutionary dynamics

These results imply that in corresponding evolutionary models utilizing direct strategy revision protocols^42^, depending on parameters and initial conditions, the system can go to either (0, 0) or (1, 1) state.

To illustrate these possibilities, assume that the subordinate always plays the best response to the leader’s previous action, i.e. $\tilde{x}=y$. Let the leader use a mixed strategy $q$. There are four possibilities for a combination of $x$ and $y$ entering the leader’s utility Eq. (7): $(0,0),(0,1),(1,0)$ and $(1,1)$ with probabilities ${(1-q)}^{2},q(1-q),q(1-q)$ and ${q}^{2}$, respectively. [Note that *x* is equal to *y* in the previous time step]. Therefore the expected utility to strategy *q*

$$\begin{array}{rcl}{U}_{L}(q) & = & {(1-q)}^{2}{u}_{L}(0,0)+q(1-q){u}_{L}(0,1)\\  &  & +\,q(1-q){u}_{L}(1,0)+{q}^{2}{u}_{L}(1,1)\\  & = & k(1-\omega )q(q-{q}^{\ast }).\end{array}$$

where ${q}^{\ast }={p}_{c}$. That is, function ${U}_{L}(q)$ is quadratic with a maximum at $q=1$.

If candidate strategies evaluated by the leader deviate only slightly from their current strategy, the dynamics will proceed in the direction of the gradient of ${U}_{L}(q)$. That is, if ${q}^{\ast } < 0$, or equivalently $\theta b > (1-\omega )(k+h)$, *q* will evolve to 1 for any initial condition. If $\theta b < (1-\omega )(k+h)$, *q* evolves to 1 if it exceeds q*/2 initially. These conclusions are not affected qualitatively if the leader make errors in predicting the subordinate’s behavior (see the SI). If candidate strategies can deviate from the current strategy substantially, reaching the state $q=1$ can happen quickly and for any initial condition. The evolution towards the state where both players always make maximum efforts is the new dynamical feature made possible by the leader’s foresight.

### Learning

Finally we compare foresight with two models of learning: reinforcement learning^40^ and payoff-biased selective imitation^41^.

#### Reinforcement learning

In reinforcement learning, agents form opinions of strategies based on the payoffs received when those strategies are implemented. Following ref. ^40^, let ${U}_{X}(x,y)$ be the utility to player $X\in L,C$ when the subordinate plays $x$ and the leader plays $y$. Let $p(t)$ be the probability the subordinate contributes and $q(t)$ be the probability the leader inspects at time step $t$. Then in Cross’ learning process^40^ version of our model the probabilities $p$ and $q$ change according to stochastic equations

10a $$p(t+1)=x{u}_{S}(x,y)+(1-{u}_{S}(x,y))p(t),$$

10b $$q(t+1)=y{u}_{L}(x,y)+(1-{u}_{L}(x,y))q(t).$$

What this means is that, after players observe how their current action (i.e. $x$ or $y$) did, they update their state (i.e., $p$ or $q$) by taking a weighted average between their old state and the state that puts all the weight on the current action (either 0 or 1), where utility ${U}_{X}(x,y)$ serves as the weight. This approach requires^40^ that all utilities are scaled to be between 0 and 1. We can achieve this, e.g. be defining them as

$${u}_{S}(x,y)=\frac{\exp \{\lambda {\pi }_{S}(x,y)\}}{{\sum }_{x}\,\exp (\lambda {\pi }_{S}(x,y))},\,{u}_{L}(x,y)=\frac{\exp \{\lambda {\pi }_{L}S(x,y)\}}{{\sum }_{y}\,\exp (\lambda {\pi }_{L}(x,y))},$$

where *λ* is a parameter. By this construction, utility increases with the payoff, and all utilities fall between 0 and 1.

In the continuous time limit, stochastic system (10) can be approximated^40,47^ by deterministic differential equations:

11a $$\frac{dp(t)}{dt}=p(t)({U}_{S}(x=1)-{\bar{U}}_{S}),$$

11b $$\frac{dq(t)}{dt}=q(t)({U}_{R}(y=1)-{\bar{U}}_{L}).$$

where

$$\begin{array}{rcl}{U}_{S}(x=1) & = & (1-q){u}_{S}(1,0)+q{u}_{S}(1,1),\\ {U}_{R}(y=1) & = & (1-p){u}_{L}(0,1)+p{u}_{L}(1,1)\end{array}$$

are the expected utilities of strategies $x=1$ and $y=1$, and

$$\begin{array}{rcl}{\bar{U}}_{S} & = & pq\,{u}_{S}(1,1)+p(1-q)\,{u}_{S}(1,0)+(1-p)q\,{u}_{S}(0,1)\\  &  & +\,(1-p)(1-q)\,{u}_{S}(0,0),\\ {\bar{U}}_{L} & = & pq\,{u}_{L}(1,1)+p(1-q)\,{u}_{L}(1,0)+(1-p)q\,{u}_{L}(0,1)\\  &  & +\,(1-p)(1-q)\,{u}_{L}(0,0)\end{array}$$

are the expected utilities of subordinates and leaders, respectively.

We analyzed both the stochastic and deterministic versions of this model. Stochastic numerical simulations of Eq. (10) show that the system always converges to equilibrium (0, 0) (see the SI). This conclusion is supported by linear stability analysis of equilibria of Eq. (11): the only stable equilibrium is (0, 0) (see the SI). We conclude that reinforcement learning is unable to overcome the free-rider-like problem in our model.

#### Selective imitation

Here we assume that individuals compare their payoff with that of a peer and choose to either copy the selected individual (if their payoff is higher than the focal individual’s) or keep their own strategy^41^.

Consider a population of pairs each consisting of a subordinate and a leader. Let the *i*-th pair’s actions at time *t* be denoted by $({x}_{i}(t),{x}_{i}(t))$ where all variable retain their usual interpretations. For each individual *i*, randomly select a peer *j* (i.e. leader for leader and subordinate for subordinate) and have the focal individual observe that peer’s payoff and action. Then the probability that at time *t* subordinate *i* continues playing their current action given they observed subordinate *j* is

$${p}_{t}(i\to i|j)=\frac{\exp \{\lambda {\pi }_{S}({x}_{i}(t),{y}_{i}(t))\}}{\exp \{\lambda {\pi }_{S}({x}_{i}(t),{y}_{i}(t))\}+\exp \{\lambda {\pi }_{S}({x}_{j}(t),{y}_{j}(t))\}},$$

which of course means the probability *i* switches to mimic *j* is ${p}_{t}(i\to j|j)=1-{p}_{t}(i\to i|j)$. A similar equation describes changes in the leader’s probability ${q}_{i}$ given they observe leader *j*′. As above, *λ* measures precision in payoff comparisons.

Assume that leaders and subordinates update their strategies at the same rate. In numerical simulations (not shown) the leaders and subordinates opt to do nothing. However if subordinates always play the best response to their leader’s previous action (i.e., if $x={y}_{prev}$), the system can evolve to a state with nonzero efforts. (See the SI for numerical illustrations).

#### Pure strategies

Consider the case of pure strategies: inspect and not inspect. Given our assumptions about parameters and best response in the subordinates, the former strategy always has a higher payoff than the latter. Therefore the frequency of leaders who inspect will always increase (subject to stochastic errors). The larger precision parameter *λ*, the faster it happens.

#### Mixed strategies

If leaders use mixed strategies, then using an approach similar to the one we applied to analysing foresight, there are four possible combinations of $x$ and $y$ in the equation for the leader’s payoff ${\pi }_{L}(x,y)$: $(0,0),(0,1),(1,0)$ and $(1,1)$ with probabilities ${(1-q)}^{2},q(1-q),q(1-q)$ and *q*^2^, respectively. Therefore the expected payoff to the leader’s strategy *q* is

$$\begin{array}{rcl}{\Pi }_{L}(q) & = & {(1-q)}^{2}{\pi }_{L}(0,0)+q(1-q){\pi }_{L}(0,1)\\  &  & +\,q(1-q){\pi }_{L}(1,0)+{q}^{2}{\pi }_{L}(1,1)\\  & = & kq(q-{q}^{\ast \ast }).\end{array}$$

where ${q}^{\ast \ast }=1-\frac{\theta b-h}{k}$. This is a quadratic maximized at $q=1$. If variation in *q* in the population is small, *q* will evolve in the direction of the gradient of ${\Pi }_{L}(q)$. That is, if the cost of punishment is small (i.e., $k < \theta b-h$), q** is negative, and ${\Pi }_{L}(q)$ is always increasing with $q\in [0,1]$. Thus, *q* is expected to evolve by selective imitation to $q=1$ for any initial value. If the cost of punishment is large (i.e., $k > \theta b-h$), then $0\le {q}^{\ast \ast }\le 1$. So *q* will increase to one for initial $q > {q}^{\ast \ast }$/2, but will decrease to zero for initial $q < {q}^{\ast \ast }$/2. If variation in *q* in the population is large, the population will always evolve towards increasing *q*.

That is, with the best response in subordinates and selective imitation in leaders the dynamics are similar to those under foresight. In both cases, the system can evolve to state $(1,1)$.

## Discussion

Here we have studied the impact of foresight on leader-subordinate dynamics in some simple models. Our aim in doing this was to shed light on what can motivate individuals to enforce contribution to production of a collective good. Typically, such enforcement comes with an inherent cost that discourages group members from being coercive as they seek to avoid the cost. This is known as the the second-order free-rider problem. Earlier work highlighted several mechanisms such as meta-punishment^12^, conformism^48^, signaling^49^, and group-selection^50,51^ as potential routes to overcoming the second-order free-rider problem. We have shown here as well as in ref. ^38^ that, in addition to these mechanisms, foresight is an effective way of motivating enforcement of cooperation. Here, “foresight” refers to a novel strategy update protocol, which stresses two key components^38^. First, that individuals care about their future payoffs. Second, that individuals consider how others will respond to their present actions in future interactions. Both of these are fairly intuitive assumptions that make few requirements of agent’s cognitive abilities. Consideration of future interactions in important in many other game-theoretic models^39,42,52–54^. By developing foresight we sought to incorporate the deterrence theory^55^ into our model, which is the notion that punishment is used to modify the future behavior of the target.

We approached this problem by altering the payoffs and assumptions of the inspector game^26^. In particular, we were concerned with modeling the interaction between a single leader interested in enforcing production and a single commoner tasked with producing some good. In this way we were able to incorporate characteristics of the general collective action problem (namely the first- and second-order free-rider effects) into a simple 2x2 game. While our earlier work^38^ has relied exclusively on numerical simulations, the simplicity of our models here has allowed us to get some analytical results.

Our models can be interpreted as describing a simple case of institutionalized punishment. There are both similarities but also differences with earlier evolutionary studies of social institutions. In our paper, the evolving part of the institution of leadership was the level of monitoring which translated into punishment levels in a way similar to that in refs. ^56,57^. In refs. ^11,58^ it was the tax imposed by the leaders while in ref. ^59^ it was the proportion of public goods invested into the group’s growth rate. In refs. ^11,58,59^ players inherited their strategies from parents (subject to rare random mutation). In refs. ^56,57^ players used payoff-biased imitation. In contrast, we have considered and compared a number of different strategy revision protocols.

We started by considering several different ways of simulating human behaviour, namely Nash equilibria, Quantal Response Equilibria, level-*k* cognition and fictitious play. Our results show that each of these methods were vulnerable to the second-order free-rider problem. That is, in these basic models while the subordinate could be motivated to produced the good, the leader was not inclined to enforce production and as a result nothing got done. We proceeded by analyzing the effect foresight had on the best response functions and the Nash equilibrium. Upon introducing foresight, we saw that the leader now viewed punishment as an utility increasing action and thus (provided sufficient emphasis on future payoffs) willingly enforced production of their subordinate. Foresight in the subordinate only served to lessen the magnitude of the their payoff. This difference in impact is due the fact that the subordinate’s action do not directly influence the leader on the same scale as the leader’s action directly influence the subordinate. Our main results are that the introduction of foresight produced new Nash equilibria at which leaders led and subordinates followed. These new equilibria were found to be dependent upon the emphasis placed on future payoffs measured by parameter $\omega$. Additionally, we found that in the repeated leadership game that foresight could effectively overcome the second-order free-rider effect. Even when error was introduced into the leader’s predictions, they were motivated to inspect provided certain conditions were met. Our final task was to compare foresight with two other strategy update protocols: reinforcement learning and selective imitation. Our results show that reinforcement learning is not able to overcome the second-order free-rider effect. In contrast, selective imitation is able to accomplish that.

Earlier refs. ^56,57^ studied similar models of institutionalized punishment with multiple subordinates per leader. They showed that selective imitation can lead to the evolution of punishment if leaders update their strategy at a much slower rate than subordinates. This happens because a low update rate prevents the leaders from abandoning a costly punishment strategy before subordinates have learned to contribute to avoid punishment. In contrast, in our model of selective imitation subordinates do not have to learn from others via incremental improvements to adapt but rather they use the best response to the current strategy of the leader. This introduces a new Nash equilibrium which can be then discovered by some leaders via random innovation and then spread across the whole system by imitation. In a similar way, foresight in leaders results in the appearance of a new Nash equilibrium discoverable by leaders via, say, a process of mental scenario building by considering several candidate strategies and comparing their expected utilities (i.e., by using a direct strategy revision protocol *sensu* ref. ^42^).

In our approach, players condition their actions on anticipated future payoffs. An alternative, which we have not explored here, is that players condition their actions on the memory of past events. For example, the leader can use a reciprocal, memory-based strategy such as “inspect with probability p if the subordinate shirked and inspect with probability q if the subordinate produced”. (We are grateful to an anonymous reviewer for suggesting this possibility). It is possible that such a strategy space will produce more Nash equilibria or ESS’s than those discussed here (see for example, refs. ^60,61^).

Our approach is related to models of level-k cognition^44,62^. Specifically, best response utilized by subordinates can be viewed as a level-1 strategy to level-0 players who do not change their strategies while foresight in leaders is related to level-2 reasoning. Typically level-k model assume that level-0 players choose their strategies uniformly randomly. Were we to make this assumption, neither inspection nor production would happen in our model. Thus, our work shows that the exact assumptions placed on the level-0 players strongly impacts the overall dynamics of the game.

Overall, our work highlights the importance of strategy revision protocols in evolutionary dynamics^42^. While the free-rider problems exists regardless of the strategy revision protocol employed, the assumptions made on how people think can impact how effective groups are at overcoming these problems. Our protocol of foresight is a new way to consider how people think, which can be used in conjuncture with existing strategy revision protocols.

There are several different questions of interest that must be answered by future work. First and foremost is the question of whether foresight would evolve in a population where it is initial absent. In our current and prior paper, we have taken for granted that foresight is present and sought only to show how it could be an effective route to overcoming the second-order free-rider problem. Having proved its efficacy we should now turn our attention to whether or not its emergence is a reasonable assumption. Secondly, here we considered the leadership game for only two players. A reasonable extension would be to assume multiple agents acting in the role of subordinates (and potential in the role of leaders as well). Thirdly, our results indicate that foresight can affect the basic dynamics of a game (in that it alters the Nash equilibria). It would be a worthwhile endeavour to investigate the impact foresight has on a wider range of classical games.

## Supplementary information

Supplementary Information.
