# Supplementary material for: Foresight in a Game of Leadership
Source: Sci Rep. 2020 Feb 10;10:2251. doi: 10.1038/s41598-020-57562-1 (PMC7010814; doi:10.1038/s41598-020-57562-1)
Supplement: Supplementary file 1 — Supplementary Information. [file 41598_2020_57562_MOESM1_ESM.pdf]

Supplementary Information for

"Foresight in a Game of Leadership"

Logan Perry and Sergey Gavrilets

Here we provide additional information on the following topics discussed in the main paper:

- Classic Inspection Game
- Quantal Response Equilibirium (QRE) solution
- Foresight for subordinate
- Phase plane analysis of the foresight model
- Errors in foresight
- Reinforcement learning dynamics
- Selective imitation dynamics

Classic Inspection Game

In the classic Inspection Game, there is a worker who works for a principal[26]. The worker can choose to shirk or work, where working costs the worker g and produces output of value v for the principal. The principal can either inspect or not inspect. The act of inspecting costs the principal h but is allows the principal to determine if the worker shirked or not. Now the principal is set to pay the worker a wage of w unless they have evidence that the worker shirked. Additional assumptions are that $w > g > h > 0$. This yields the game as described in Table S1. This game has no pure-strategy equilibrium, but does have a mixed Nash equilibrium at $(x,y) = \left(\frac{w-h}{w}, \frac{g}{w}\right)$, where x is the probability that the worker shirks, and y is the probability that the principal inspects.

		Principal	
		Inspect	Don't Inspect
Worker	Works	$w-g,\ v-w-h$	$w-g,\ v-w$
	Shirks	$0,\ -h$	$w,\ -w$

Table S1. Payoff matrix for the classic Inspection Game[26].

Quantum Response equilibrium (QRE) solution

Figure S1 shows the impact of the precision parameter λ on the QRE values (p^*, q^*) as found numerically by solving equation () of the main text. For $\lambda = 0$, play is perfectly random so that $p^* = q^* = \frac{1}{2}$, as we would expect. As we increase λ, play converges to the single Nash equilibrium at $(0,0)$, again as we would expect.

Foresight for subordinate

Assume that the subordinates cares about the future payoff and predicts that the leader will use the best response. Because the leader's best response is always 0, the subordinate predicts that there will be no monitoring and punishment and thus will choose not to contribute.

Phase plane analysis in the foresight model

Let p and q be the probabilities of making an effort (i.e., the mixed strategies) for the subordinate and leader. Their expected utilities are

$$U_S = ((1-\theta)b - c)p - dq(1-p) \tag{S1a}$$
$$U_L = (1-\omega)(-h + k(1-p))q) + \omega\theta bq \tag{S1b}$$

This corresponding derivatives are given by

$$\partial_p U_S = ((1-\theta)b - c) + dq \tag{S2a}$$
$$\partial_q U_L = (1-\omega)(-(h + k(1-p))) + \omega\theta b. \tag{S2b}$$

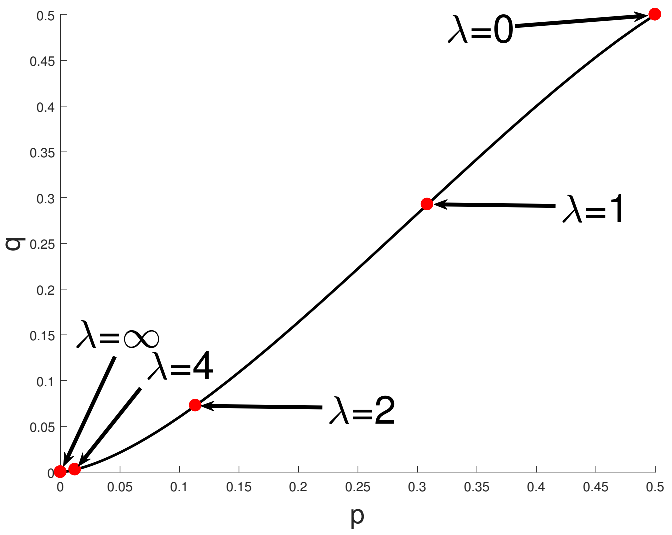

Figure S1. Effect of the precision parameter λ on the Quantal Response Equilibrium values p^* and q^*. Parameters: $b = 1, c = 2, d = 2, h = 0.1, k = 1$, and $\theta = 0.5$.

As discussed in the main text, $\partial_q U_L < 0$ whenever $p < q^{**} = 1 + \frac{1}{k}\left(h - \frac{\omega\theta b}{1-\omega}\right)$, and $\partial_q U_L > 0$ whenever $p > q^{**}$, and $\partial_p u_S < 0$ whenever $q < q_c = \frac{c-(1-\theta)b}{d}$ and $\partial_p u_S > 0$ whenever $q > q_c$. This yields the expected flow on the phase plane (p,q) as outlined in Figure S2.

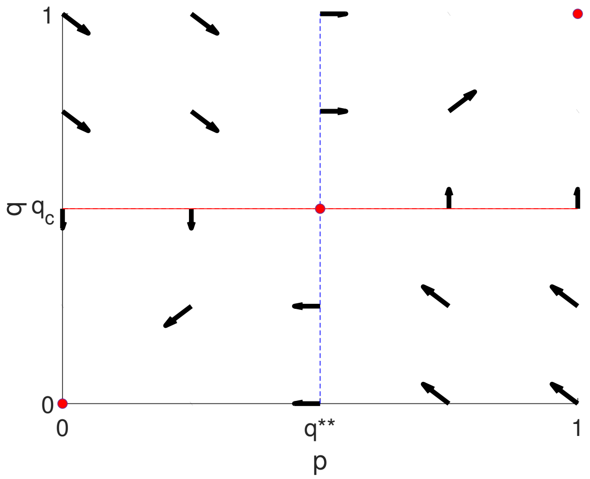

Figure S2. Flow associated with equations (S1a. Parameters: $b = 1, c = 2, d = 3, k = 1, h = .25, \theta = 0.5$, and $\omega = 0.6$. Red dots mark Nash equilibria two of which are stable $(0,0$ and $(1,1))$ and one unstable at (q^{**}, q_v).

Errors in foresight

Here we incorporate errors into the leader's predictions of the actions of the subordinate. We can interpret this error in one of two ways. First, it can be that the leader makes mistakes when attempting to decide what the subordinate will do. Second, it can be that the leader anticipates that the subordinate will make mistakes and takes this into account. Either way, we define the probability $p(y)$ the leader will predict the subordinate contributes given the leader inspects with probability q as

$$p(y) = \frac{1}{1 + \exp[\lambda(\pi_S(0,y) - \pi_S(1,y))]} = \frac{1}{1 + \exp[\lambda(-dy - b(1-\theta) + c]}.$$

That is, we allow for logit errors with precision parameter λ as above.

Using the same approach as in the main text, we write the leader's utility as

$$U_L(y_{prev}, y) = (1-\omega) \times (-[h + k(1 - p(y_{prev}))]y) + \omega \times (\theta b p(y))$$

where y_{prev} and y are two subsequent values of the leader's y. Then, for a leader using strategy q, the expected utility is

$$U(q) = (1-q)^2 U_L(0,0) + q(1-q)U_L(0,1) + q(1-q)U_L(1,0) + q^2 U_L(1,1).$$

From the above equation we can find the critical value q^* such that for initial values of q above q^*, q is expected to evolve to 1. Figure S3 illustrate the dependence of q^* on λ and ω. This Figure shows that increases ω increases decreases q^* and thus increases the range of conditions leading to production and inspection. As $\lambda \to \infty$, the predicted value of q^* converges to that one found in the main text.

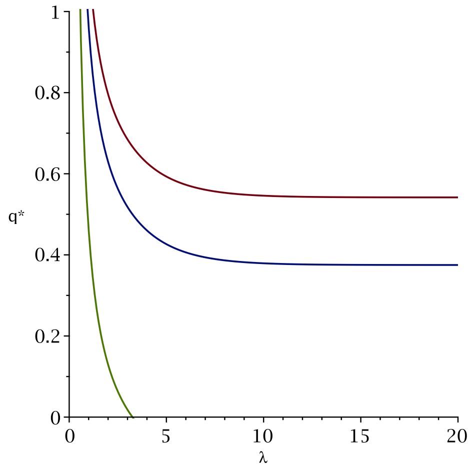

Figure S3. The critical value q^* in the model with errors in the leader's foresight for different values of precision parameter λ. The three curves correspond to $0 = 0.75, 0.5$ and 0.25 (from top to bottom). Other parameters: $b = 1, c = 2, d = 2, h = 0.25, k = 1$, and $\theta = 0.5$.

Reinforcement learning dynamics

The system of differential equations (11) in the main text has four equilibria: $(0,0), (1,0), (0,1)$ and $(1,1)$. Standard stability analysis shows that the only locally stable equilibrium is $(0,0)$. For example, the eigenvalues of the Jacobian matrix at this state are $u_S(1,0) - u_S(0,0)$ and $u_L(0,1) - u_L(0,0)$, which are always negative by our assumptions. The corresponding eigenvalues of state $(1,1)$ are $u_S(0,1) - u_S(1,1)$ and $u_L(1,0) - u_L(1,1)$, which are always positive by our assumptions.

Numerical simulations show that the system may spend very long time in a neighborhood of each of the other three equilibria, that is, the dynamics are characterized by long transients[63]. For example, Figure S4 shows the stochastic trajectories in the reinforcement learning model for a range of random initial conditions. With small precision λ, fluctuations in p and q persist for a long time. Even with large λ some subordinate-leader pairs can stay close to state $(1,1)$ for a long time.

Selective Imitation

Consider first the case where both leaders and subordinates update their actions x and y via selective imitation. Figure S5 shows the trajectories associated with a range of random initial conditions drawn from the uniform distribution over $[0.1, 0.9]$. We see that regardless of the initial condition, the system eventually settles into the equilibrium of $(0,0)$.

Next consider the case where leaders update via selective imitation, but subordinates update via myopic optimization. Figure S6 shows the trajectories associated with a range of random initial conditions drawn from the uniform distribution over $[0.1, 0.9]$. With small precision λ the system may vocationally end up at $(0,0)$ state. [This is analogous to fixing a deleterious allele by random genetic drift in population genetics.] With increasing precision λ, the system always evolves to $(1,1)$ state.

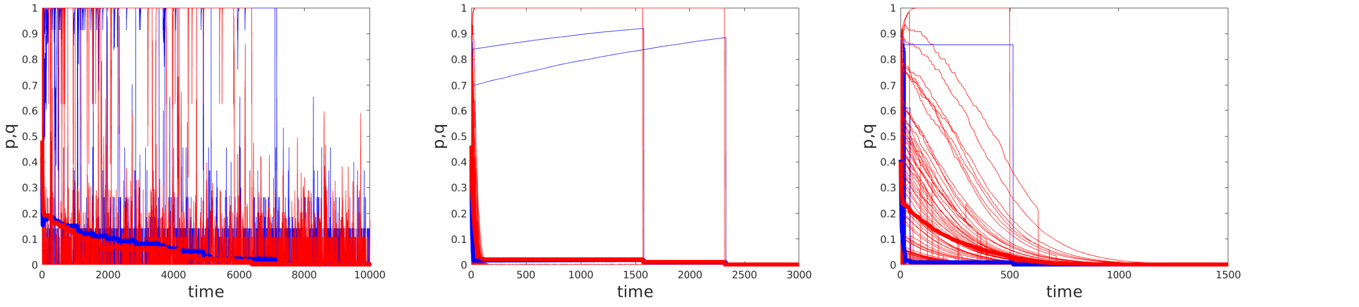

(a) $\lambda = 1$ (b) $\lambda = 5$ (c) $\lambda = 10$

Figure S4. Time-series in the case of reinforcement learning for different values of λ. The graphs show 100 independent runs with initial conditions drawn from the uniform distribution over $[.1, .9]$. The thin lines represent the individual runs while the thick lines represent the mean over all runs with the red corresponding the the leader and the blue to the subordinate. Parameters: $b = 1, c = 2, d = 2, k = .5, h = .25$, and $\theta = 0.5$.

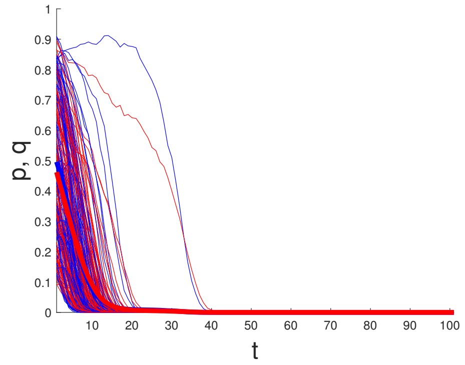

Figure S5. Time-series in the case of selective imitation in both players. The thin lines represent the individual runs while the thick lines represent the mean over all runs with the red corresponding the the leader and the blue to the subordinate. Note that all trajectories tend toward the equilibrium (0,0). Parameters: $b = 1, c = 2, d = 2, k = .5, h = .25, \theta = 0.5$, and $\lambda = 1$.

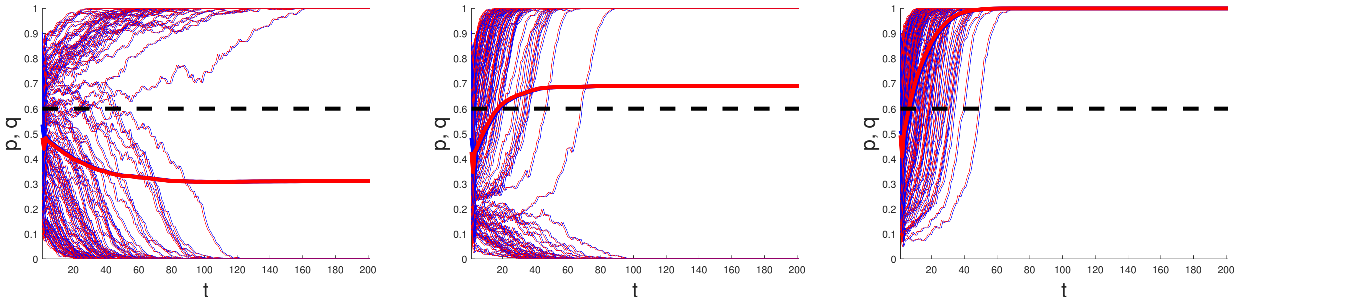

(a) $\lambda = 1$ (b) $\lambda = 5$ (c) $\lambda = 10$

Figure S6. Time-series in the case of myopic optimization in the subordinate and selective imitation in the leader for different values of λ. The results summarize 100 independent runs with initial conditions drawn from the uniform distribution over $[0.1, 0.9]$. The thin lines represent the average over all 1000 groups while the thick lines represent the mean over all runs with the red corresponding the the leader and the blue to the subordinate. Parameters: $b = 1, c = 2, d = 2, k = .5, h = .25$, and $\theta = 0.5$.
